# Incidence of foodborne diseases in Ecuador

**DOI:** 10.17843/rpmesp.2024.413.13456

**Published:** 2024-09-03

**Authors:** Angelica Ochoa-Avilés, Samuel Escandón, Cristina Ochoa-Avilés, Odalys Heredia-Andino, Johana Ortiz-Ulloa

**Affiliations:** 1 Food, Nutrition, Health and Physical Activity Research Group, Department of Biosciences, Faculty of Chemical Sciences, University of Cuenca, Cuenca, Ecuador. University of Cuenca Food, Nutrition, Health and Physical Activity Research Group, Department of Biosciences Faculty of Chemical Sciences University of Cuenca Cuenca Ecuador

**Keywords:** Foodborne Illness, Food Safety, Incidence, Public Health, Epidemiology

## Abstract

In order to describe the incidence rates per 100 000 population of foodborne disease (FBD) cases during the period 2015-2020 in Ecuador, we carried out a secondary analysis of epidemiological surveillance records and population projections from the National Institute of Statistics and Census. A total of 113,695 cases were reported with an incidence of more than 100 cases per 100 000 population (2015-2019). In 2020, the records are considerably lower than those reported in previous years. Most cases were reported as “other food poisoning”. The highest incidence rates of FBD were found in the Amazon region. In general, there is a marked annual variability in the incidence of FBD according to the geographic regions of Ecuador. In conclusion, FBD represent a public health problem in Ecuador. Comprehensive preventive strategies should be designed with special emphasis on the Amazon region.

## INTRODUCTION

Poor food safety is a major public health problem worldwide. Microbiological, chemical, and physical hazards can lead to food safety problems throughout the supply chain ^1^. Foodborne diseases (FBD) pose health threats and represent a high cost to health services ^2^^,^^3^. In areas where sanitary conditions are poor or there are problems with the food supply chain, morbidity and mortality rates related to FBD remain high. According to a 2015 World Health Organization (WHO) report, unsafe food was responsible for 600 million cases of FBD and 420,000 deaths per year, accounting for about 33 million years of life lost globally ^4^.

The impact of FBD is greater among children living in low-income regions where food hygiene and water sanitation are below optimal standards ^3^. The types of FBD, their severity, and their impact have changed over the years and differ among age groups, regions, and countries ^5^. It is essential to have epidemiological surveillance systems that allow the development of prevention, monitoring and intervention strategies and policies ^2^. Along with monitoring and surveillance, voluntary reporting is part of risk management, as it helps to identify problems and ensure a safe food supply ^3^.

In Ecuador, the Ministry of Public Health (MPH), through the National Subsecretariat of Public Health Surveillance and the National Directorate of Epidemiological Surveillance, has implemented the Integrated Epidemiological Surveillance System (SIVE) ^6^. Although the SIVE is a major advance regarding food safety, it only reports net data on cases per year and per province without estimating population-adjusted incidence rates ^7^^,^^8^. The only published study is limited to a single pathogen (hepatitis A), in a specific year ^9^, without calculating figures by year and geographical region adjusted for population size.

This study aimed to describe the incidence rates of FBD cases registered in the Ecuadorian MPH SIVE during 2015-2020 in the regions and provinces of mainland Ecuador.

**KEY MESSAGES**

Motivation for the study. In Ecuador, foodborne disease (FBD) incidence rates adjusted for population size have not been estimated, which will serve to identify priority geographic areas.

Main findings. Between 2015-2020, 113,695 cases of FBD were identified, with “other food poisoning” and hepatitis A being most common. The highest incidence rates were found in the Amazon region. There is marked variability by geographic region in the incidence rates during the study period.

Public health implications. It is necessary to optimize the registry system, establish detection and treatment protocols, analyze the causes related to the higher incidence of FBD in the Amazon region, and design a health promotion program focused on preventing contamination and establishing diagnostic and treatment protocols.

## THE STUDY

The study area included continental Ecuador, which is made up of three regions with different climates and altitudes: the Sierra with 10 provinces, the Coast with 7 provinces, and the Amazon with 6 provinces. In 2020, the population of continental Ecuador was estimated at 17,510,643 inhabitants ^10^.

Due to changes in the Surveillance System of the National Directorate of Epidemiological Surveillance of the MPH of Ecuador, the information on FBD cases was compiled from the epidemiological “Ecuador SIVE-ALERT” gazettes for the period 2015 to 2018 ^7^^)^ and from the “Toxic Effects” gazettes for the period 2019 and 2020 ^8^. FBD cases are registered by the medical staff of health institutions through the EPIC2 form ^7^. The number of annual cases between 2015 and 2020 of typhoid and paratyphoid fever, hepatitis A, salmonellosis, shigellosis and, so-called “other bacterial food poisoning” were calculated from weekly reports from the SIVE website. This website does not specify the causative agents included in the category “other bacterial food poisoning” classified in ICD-10 with code A05.

Weekly report data from the MPH gazettes were entered into an Excel spreadsheet. Verification of each data was performed by a second investigator (COA). The weekly cases for each year from 2015 through 2020 were summed to obtain the reported cases during each year. There were inconsistencies when comparing the weekly records with the cumulative value presented in SIVE, so we decided to present the annual cumulative value of the last week of registration, under the suspicion of delays in the weekly reports. The sum of the total number of cases registered during the six years of analysis, the average and median number of cases during the six analyzed years are presented.

Incidence rates were estimated per 100,000 inhabitants according to the intercensal projections of the National Institute of Statistics and Census (INEC) for each geographic region (Coast, Highlands and Amazon) and for each province of continental Ecuador according to the year of registration (2015-2020) ^11^. We used data from INEC official intercensal projections, which are estimated based on the extrapolation of historical trends and on hypotheses of future behavior of fertility, mortality and migration, being valid methods for the periods between censuses (The census scheduled for 2020 was postponed due to the COVID-19 pandemic) ^12^. The incidence rate per 100,000 inhabitants per year is presented for each FBD (typhoid and paratyphoid fever, hepatitis A, salmonellosis, shigellosis, and other food poisoning).

The incidences of each FBD, the sum of the incidence of all analyzed FBD per 100,000 according to geographic region (Coast, Highlands and Amazon) and year of registration, are reported in bar graphs. The incidences of the sum of all the FBD per 100,000 inhabitants according to the year of registration and the provinces of continental Ecuador are presented in geographical distribution maps.

Data analysis was performed with the RStudio 4.3.3 program (RStudio Team [2024]. RStudio: Integrated Development for R. RStudio, PBC, Boston, MA URL http://www.rstudio.com/). The maps were produced using the Datawrapper web applications (https://www.datawrapper.de).

Considering that the study used anonymized, publicly available secondary data, approval by a human research ethics committee was not required.

## FINDINGS

**Figure 1:**
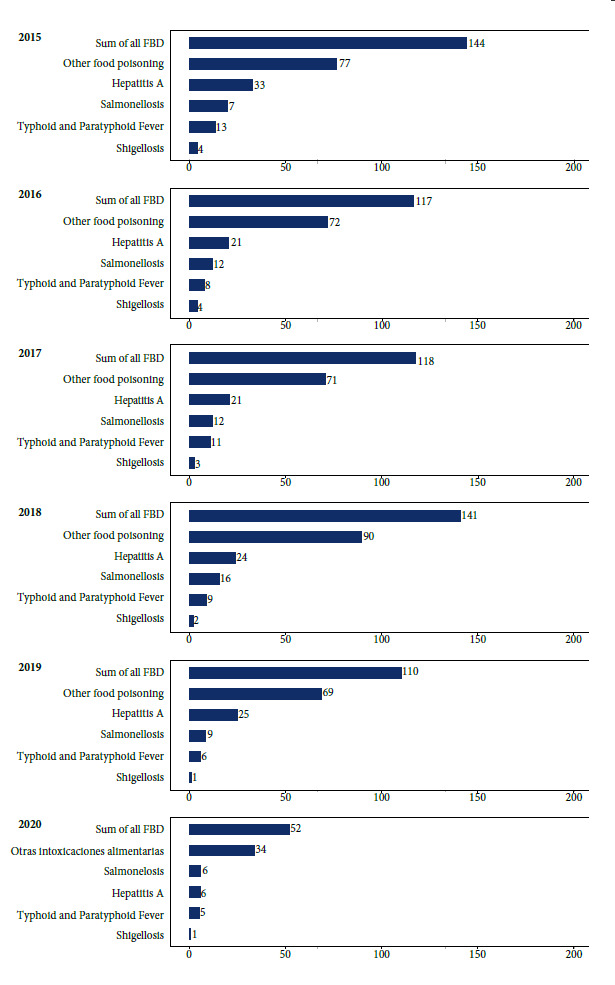
Incidence of FBD per 100,000 population in continental Ecuador by year (period 2015-2020).

Table 1 shows the net number of FBD registrations per year and Figure 1 presents the incidence per 100,000 population nationwide. There were 113,695 cases with an incidence of more than 100 cases per 100,000 population (except in 2020). Most cases are reported as “other food poisoning”, i.e. the causative agent is not reported in most cases. This is followed by hepatitis A, salmonellosis, typhoid fever and shigellosis. The lowest number of FBD cases was reported in 2020.

**Table 1:** Frequency of foodborne diseases in mainland Ecuador between 2015 and 2020 ^a^.

	2015	2016	2017	2018	2019	2020	Total	Mean (SD)	Median
Other food poisoning ^b^	12,347	11,790	11,921	15,397	11,855	5890	69,200	11,533 (3090)	11,888
Hepatitis A	5355	3399	3502	4146	4224	1057	21,683	5355 (1434)	3824
Salmonellosis	2732	1893	2041	2647	1546	1099	11,958	2732 (630)	1967
Typhoid and Paratyphoid Fever	2087	1241	1709	1515	1059	766	8377	1396 (474)	1378
Shigellosis	553	627	562	387	238	110	2477	413 (205)	470

a Data calculated from reports from the surveillance system of the Ministry of Public Health of Ecuador.

b The surveillance system does not specify the causal agents included in the category “Other bacterial food poisoning” classified in the ICD-10 with code A05.

**Figure 2:**
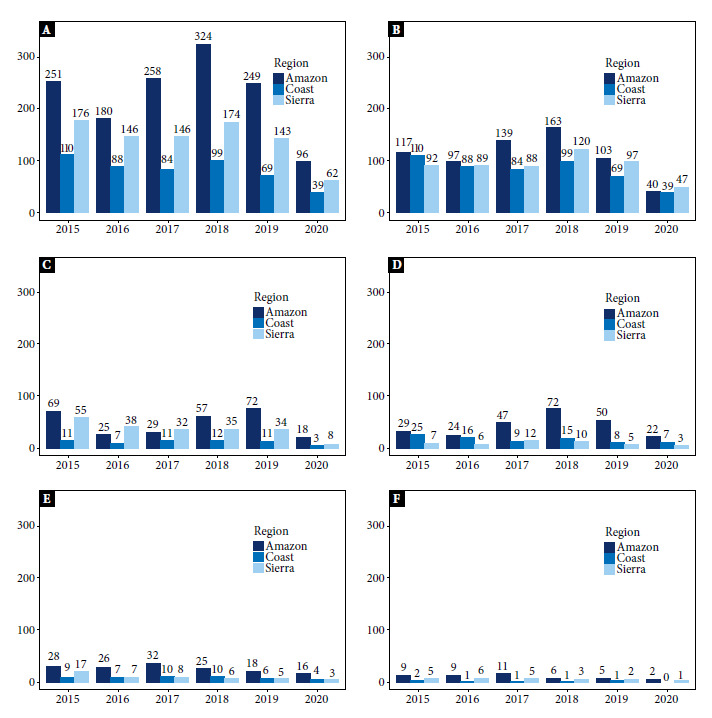
Incidence of FBD per 100,000 population by geographic region (Coast, Highlands and Amazon) and year (2015-2020). A) Sum of all FBD, B) Other food poisoning, C) Hepatitis A, D) Salmonellosis, E) Typhoid and Paratyphoid Fever and F) Shigellosis.

Figure 2A shows the incidence rates per 100,000 inhabitants by geographic region of Ecuador. The Amazon region has the highest incidence rate (above 180 cases per 100,000 inhabitants between 2015-2019), followed by the Sierra region and the Coast region. A higher number of cases was reported in 2018.

In the Amazon region, hepatitis A had the highest incidence rates in the years 2015, 2018, 2019 and 2020, reaching 72 cases per 100,000 inhabitants in 2019 (Figure 2C). For its part, salmonellosis presented the highest incidence in the years 2017 and 2018, affecting 72 patients per 100,000 inhabitants in 2018 (Figure 2D). In the Sierra, hepatitis A had the highest incidence rate, with more than 30 cases of per 100,000 inhabitants in the years 2015-2019 (Figure 2C), while none of the other FBD with identified causative agent exceeded 17 cases per 100,000 inhabitants (Figure 2). The predominance of hepatitis A observed in the Sierra region was not replicated in the Costa region, where salmonellosis exceeded hepatitis A during 2015, 2016, 2018 and 2020 (Figures 2C and D). Excluding food poisoning without a defined causative agent, none of the analyzed FBD exceeded 25 cases per 100,000 population in the Coast region (Figure 2). Regarding the incidence of typhoid and paratyphoid fever from 2015 to 2020, the highest incidence rate was again found in the Amazon region, with 2017 being the year with the highest number of cases (Figure 2E).

**Figure 3:**
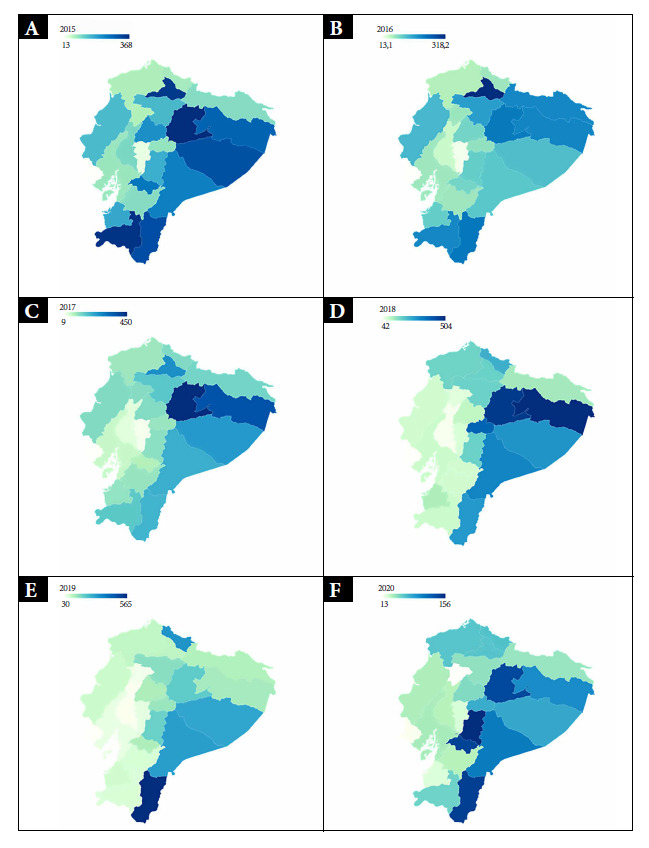
Incidence of the sum of all FBD cases per 100,000 population by province between 2015 and 2020.

The incidence rate of the sum of all FBD by province for the period 2015-2020 is presented in Figure 3. There is marked variability over the years between different provinces; the provinces responsible for the differences at the regional level can be identified. The Amazonian provinces of Napo and Orellana consistently presented the highest rates per 100,000 inhabitants in the period 2015-2018. In the years 2019 and 2020, Zamora Chinchipe presented the highest incidence rates in the Amazon region. Between 2015 and 2017, the province of Imbabura had the highest incidence of FBD in the Sierra region, while the province of Manabí had the highest incidence in the Coast region in the same period (2015-2017).

A. Sum of all FBD; B. Other food poisoning; C. Hepatitis A; D. Salmonellosis; E: Typhoid and paratyphoid fever; F. Shigellosis.

## DISCUSSION

This study systematized the incidence of FBD reported in the surveillance system of the Ecuadorian MPH during the period 2015-2020. Despite the high rates, the problem could have been more severe considering that many cases do not seek medical attention and that the causative agent is not always detected in those that do ^4^. Underreporting affects the understanding of the problem, and thus the actions of health institutions ^4^. To ensure food safety and prevent FBD, rapid and accurate detection of pathogens is essential ^3^.

In Ecuador, there are no protocols for the detection and treatment of FBD, therefore, deficiencies related to the diagnosis and identification of the causal agents may exist, explaining the high variability of incidence rates over the years. There is no evidence that epidemiological surveillance data in Ecuador are used to design prevention strategies. The implementation of protocols for detection, treatment and follow-up is needed to ensure timely actions to counteract outbreaks, which are potentially fatal for vulnerable populations ^4^^,^^13^. Although epidemiological surveillance is a fundamental pillar, effective prevention can be achieved if essential elements converge at different levels of control and involvement of society, laws and regulations, food handling control, inspection services, laboratory services, food safety monitoring, and epidemiological surveillance supported by constant education, communication, and training of food handlers ^14^^,^^15^.

Overall, a higher incidence of FBD was consistently reported in the Amazon, which may be explained by the high rate of multidimensional poverty in the region, where in 2020, Amazonian provinces such as Napo (82.5%), Pastaza (80.1%) and Morona Santiago (81.5%) had significantly higher rates of poverty than highland provinces such as Azuay (25.6%) and Pichincha (16.9%) ^16^^-^^18^. On the other hand, ethnic minorities of lower economic strata are more affected by FBD ^13^ due to inadequate knowledge, attitudes and practices, and poor food handling practices.

Comparison of the collected epidemiological information with data from countries with similar contexts is complex due to the potential underreporting of cases ^2^. Regarding typhoid and paratyphoid fever, a study conducted in Mexico during 2018 reported an incidence three times higher (27.9%) compared to what was found in Ecuador (8.9%) ^19^. On the other hand, a study in the Department of Casanare in Colombia with low poverty rates and humid climate, similar to the Amazon, reported incidence rates of hepatitis A similar to ours in some municipalities ^20^, while compared to Chile, incidence rates in Ecuador were considerably higher (17 cases per 100,000 inhabitants in 2018 in Chile, vs. 104 cases per 100,000 inhabitants in Ecuador) ^21^. This demonstrates the difficulty in comparing data in middle- and low-income regions due to problems in reporting and timely medical care within the health system ^22^.

Most cases are reported as “other bacterial food poisoning” reinforcing the idea that the causative agents are not identified in most patients. On the other hand, in many cases the reports accumulated week by week do not correspond with the data published in previous reports, indicating the possibility that in some cases the report was delayed in the National Surveillance System. These shortcomings are not new; evidence shows that only a few countries in the world have succeeded in having a reliable and well-documented registry of FBD. Even in countries with an adequate registration system, many diseases go untreated because symptoms are mild in most cases ^22^.

The calculated incidence of hepatitis A in Ecuador is high ^23^^,^^24^. In adolescents and adults, hepatitis A can cause alteration of liver function markers, general malaise, vomiting, anorexia and in some cases death (2%) ^23^. In addition, patients may take weeks or months to recover and resume their daily activities, negatively influencing the economy and productivity ^19^. WHO has estimated the cost of total productivity loss in low- and middle-income countries at $95.2 billion annually, while the cost of treating FBD has been estimated at $15 billion ^25^.

This study has some limitations, such as the fact that we identified discrepancies in the values presented weekly with the cumulative values, so we used the cumulative values recorded in the last epidemiological week of each year. This made it difficult to achieve greater accuracy in the calculation of incidences or to analyze the data week by week.

Our analysis highlights the high incidence rates of FBD, and there is a marked annual variability in the incidence of FBD in the different geographical regions of Ecuador. It can be concluded that FBD represent a public health problem in Ecuador that mostly affects the Amazon region. It is necessary to evaluate and optimize the registration of cases and the surveillance system, as well as to design comprehensive preventive strategies with special emphasis on the Amazon region.
