# Supplementary material for: Incidence of foodborne diseases in Ecuador
Source: Rev Peru Med Exp Salud Publica. 2024 Sep 3;41(3):273–80. doi: 10.17843/rpmesp.2024.413.13456 (PMC11495946; doi:10.17843/rpmesp.2024.413.13456)
Supplement: Translation

# Translation: Incidencia de las enfermedades transmitidas por alimentos en Ecuador

**Authors:** Angelica Ochoa-Avilés, Samuel Escandón, Cristina Ochoa-Avilés, Odalys Heredia-Andino, Johana Ortiz-Ulloa

**Affiliations:** 1 Grupo de Investigación Alimentación, Nutrición, Salud y Actividad Física, Departamento de Biociencias, Facultad de Ciencias Químicas, Universidad de Cuenca, Cuenca, Ecuador.

**Keywords:** Enfermedades Transmitidas por Alimentos, Inocuidad Alimentaria, Incidencia, Salud Pública, Epidemiología

## Abstract

Con el objetivo de describir las tasas de incidencia por cada 100 000 habitantes de los casos de enfermedades transmitidas por alimentos (ETA) durante el periodo 2015-2020 en Ecuador se realizó un análisis secundario de los registros de vigilancia epidemiológica y de las proyecciones poblacionales del Instituto Nacional de Estadística y Censos. Se reportaron 113 695 casos con una incidencia superior a los 100 casos por cada 100 000 habitantes (2015-2019). En el 2020 los registros son considerablemente inferiores a los reportes de años anteriores. La mayoría de los casos se reportaron como «otras intoxicaciones alimentarias». Las tasas de incidencia de ETA más altas se observaron en la región amazónica. En general existe una marcada variabilidad anual en la incidencia de las ETA según las regiones geográficas del Ecuador. En conclusión, las ETA representan un problema de salud pública en el Ecuador. Se deben diseñar estrategias preventivas integrales con especial énfasis en la región amazónica.

## INTRODUCCIÓN

La inocuidad alimentaria deficiente es un importante problema de salud pública a nivel mundial. Peligros microbiológicos, químicos y físicos pueden generar problemas de inocuidad de los alimentos a lo largo de la cadena de suministro ^1^. Las enfermedades transmitidas por alimentos (ETA) plantean amenazas para la salud y representan un alto costo para los servicios de salud ^2^^,^^3^. En áreas donde las condiciones sanitarias son deficientes o existen problemas con la cadena de suministro de alimentos, las tasas de morbilidad y mortalidad relacionadas a ETA siguen siendo altas. Según un reporte de La Organización Mundial de la Salud (OMS) del 2015, los alimentos no inocuos fueron responsables de 600 millones de casos de ETA y 420 000 muertes por año, representando alrededor de 33 millones de años de vida perdidos a nivel global ^4^.

El impacto de las ETA es mayor entre los niños que residen en regiones de bajos ingresos donde la higiene de los alimentos y el saneamiento del agua están por debajo de los estándares óptimos ^3^. Los tipos de ETA, su gravedad y su impacto han cambiado a lo largo de los años y difieren entre grupos etarios, regiones y países ^5^. Es indispensable contar con sistemas de vigilancia epidemiológica que permitan plantear estrategias y políticas de prevención, monitoreo e intervención ^2^. Junto con el monitoreo y la vigilancia, el reporte voluntario es parte de la gestión de riesgos ya que permite identificar los problemas y garantizar el suministro de alimentos inocuos ^3^.

En Ecuador, el Ministerio de Salud Pública (MSP), a través de la Subsecretaría Nacional de Vigilancia de la Salud Pública y la Dirección Nacional de Vigilancia Epidemiológica, ha implementado el Sistema Integrado de Vigilancia Epidemiológica (SIVE) ^6^. Si bien, el SIVE es un gran avance en torno a la inocuidad alimentaria, solamente reporta los datos netos de casos por año y por provincia sin estimar tasas de incidencia ajustadas a la población ^7^^,^^8^. El único estudio publicado se ha limitado a un solo agente patógeno (hepatitis A), en un año específico ^9^, sin calcular cifras por años y regiones geográficas ajustadas para el tamaño de la población.

El objetivo de este estudio fue describir las tasas de incidencia de los casos de ETA registrados en el SIVE de MSP del Ecuador durante el 2015-2020 en las regiones y provincias de Ecuador continental.

**MENSAJES CLAVE**

Motivación para realizar el estudio. En Ecuador, no se ha estimado las tasas de incidencia de enfermedades transmitidas por alimentos (ETA) ajustadas al tamaño de la población, lo que servirá para identificar áreas geográficas prioritarias.

Principales hallazgos. Entre 2015-2020 se identificaron 113 695 casos de ETA, siendo más comunes las «otras intoxicaciones alimentarias» y la hepatitis A. Las tasas de incidencia más altas se observaron en la región amazónica. Existe una marcada variabilidad por región geográfica en las tasas de incidencia reportadas durante el periodo de estudio.

Implicancias en la salud pública. Es necesario optimizar el sistema de registro, instaurar protocolos de detección y tratamiento, así como analizar las causas relacionadas con la mayor incidencia de ETA en la región amazónica y diseñar un programa de promoción de salud enfocado en prevenir contaminación y establecer protocolos de diagnóstico y tratamientos.

## EL ESTUDIO

Se realizó un análisis de fuentes secundarias, cuya área de estudio incluyó Ecuador continental, conformado por tres regiones con clima y altitud diferentes: la Sierra con 10 provincias, la Costa con 7 provincias, y la Amazonia con 6 provincias. En el 2020, la población de Ecuador continental se estimó en 17 510 643 habitantes ^10^.

Debido a cambios en el Sistema de Vigilancia de la Dirección Nacional de Vigilancia Epidemiológica del MSP de Ecuador, la información de los casos de ETA fue recopilada de las gacetas epidemiológicas «Ecuador SIVE-ALERTA» para el periodo 2015 al 2018 ^7^ y de las gacetas «Efectos tóxicos» para el periodo 2019 y 2020 ^8^. Los casos de ETA son registrados por el personal médico de las instituciones de salud a través del formulario EPIC2 ^7^. El número de casos anuales entre el 2015 y 2020 de fiebre tifoidea y paratifoidea, hepatitis A, salmonelosis, shigelosis y, las denominadas «otras intoxicaciones alimentarias bacterianas» se calcularon de los reportes semanales de la página web del SIVE. Este sitio web no especifica los agentes causales incluidos en la categoría «otras intoxicaciones alimentarias bacterianas» clasificada en el CIE-10 con el código A05.

Los datos de reportes semanales de las gacetas del MSP fueron ingresados en una hoja cálculo de Excel. La verificación de cada dato fue realizada por un segundo investigador (COA). Los casos semanales para cada año desde el 2015 hasta el 2020 fueron sumados para obtener los casos reportados en cada año. Al comparar los registros semanales con el valor acumulado presentado en el SIVE, hubo inconsistencias, por lo que se optó por presentar el valor acumulado anual de la última semana de registro, bajo la sospecha de retrasos en los reportes semanales. Se presenta la sumatoria del total de casos registrados durante los seis años de análisis, el promedio y la mediana de casos en los seis años analizados.

Las tasas de incidencia se estimaron por cada 100 000 habitantes según las proyecciones intercensales del Instituto Nacional de Estadística y Censos (INEC) para cada región geográfica (Costa, Sierra y Amazonía) y para cada provincia del Ecuador continental según el año de registro (2015-2020) ^11^. Se utilizaron los datos de proyecciones intercensales oficiales del INEC que se estiman a partir de la extrapolación de tendencias históricas observadas y en hipótesis de comportamiento futuro de la fecundidad, mortalidad y migración, siendo métodos válidos para los perídos entre censos (El censo programado para el 2020 fue postergado debido a la pandemia de la COVID-19) ^12^. Se presenta la tasa de incidencia por cada 100 000 habitantes por año para cada ETA (fiebre tifoidea y paratifoidea, hepatitis A, salmonelosis, shigelosis, y otras intoxicaciones alimentarias).

Las incidencias de cada ETA, la sumatoria de la incidencia de todas las ETA analizadas por cada 100 000 según la región geográfica (Costa, Sierra y Amazonía) y el año de registro, se reportaron mediante gráficos de barras. Las incidencias de la sumatoria de todas las ETA por cada 100 000 habitantes según el año de registro y las provincias del Ecuador continental se presentaron en mapas de distribución geográfica.

El análisis de datos se realizó con el programa RStudio 4.3.3 (RStudio Team [2024]. RStudio: Integrated Development for R. RStudio, PBC, Boston, MA URLhttp://www.rstudio.com/*)*. Los mapas se elaboraron a través de las aplicaciones web Datawrapper (https://www.datawrapper.de).

Considerando que el estudio utilizó datos secundarios anónimos de acceso público, no fue necesaria la aprobación de un comité de ética en investigación en seres humanos.

## HALLAZGOS

La tabla 1 muestra el número neto de registros de ETA por año y la figura 1 la incidencia por cada 100 000 habitantes en todo el país. Se registraron 113 695 casos con una incidencia superior a los 100 casos por cada 100 000 habitantes (excepto en el 2020). La mayoría de los casos son reportados como «otras intoxicaciones alimentarias», es decir que en la mayoría de los casos no se reporta el agente causal. Le sigue hepatitis A, salmonelosis, la fiebre tifoidea y la shigelosis. En el año 2020 ocurre el menor reporte de casos para todas las ETA.

**Tabla 1:** Frecuencia de enfermedades transmitidas por alimentos en Ecuador continental entre el 2015 y el 2020 ^a^.

	2015	2016	2017	2018	2019	2020	Total	Promedio (DE)	Mediana
Otras intoxicaciones alimentarias ^b^	12347	11790	11921	15397	11855	5890	69200	11533 (3090)	11888
Hepatitis A	5355	3399	3502	4146	4224	1057	21683	5355 (1434)	3824
Salmonelosis	2732	1893	2041	2647	1546	1099	11958	2732 (630)	1967
Fiebre Tifoidea y Paratifoidea	2087	1241	1709	1515	1059	766	8377	1396 (474)	1378
Shigelosis	553	627	562	387	238	110	2477	413 (205)	470

a Datos calculados a partir de los reportes del sistema de vigilancia del Ministerio de Salud Pública del Ecuador

b En el sistema de vigilancia no se especifica los agentes causales incluidos en la categoría «Otras intoxicaciones alimentarias bacterianas» clasificada en el CIE-10 con el código A05.

**Figura 1:** Incidencia de ETAs por cada 100.000 habitantes en Ecuador continental por año (período 2015-2020).

La figura 2A muestra las tasas de incidencia por 100 000 habitantes por región geográfica de Ecuador. La región amazónica presenta la mayor tasa de incidencia (superior a los 180 casos por cada 100 000 habitantes entre el 2015-2019), seguida de la región Sierra y la región Costa. Se observa un mayor reporte de casos en el año 2018.

**Figura 2:** Incidencia de ETA por cada 100 000 habitantes según la región geográfica (Costa, Sierra y Amazonía) y año (2015-2020). A) Suma de todas las ETA, B) Otras intoxicaciones alimentarias, C) Hepatitis A, D) Salmonelosis, E) Fiebre Tifoidea y Paratifoidea y F) Shigelosis.

En la región amazónica, la hepatitis A, presentó la mayor incidencia en los años 2015, 2018, 2019 y 2020, alcanzando los 72 casos por cada 100 000 habitantes en el 2019 (figura 2C). Por su parte, la salmonelosis presentó la incidencia más alta en los años 2017 y 2018, afectando a 72 pacientes de cada 100 000 habitantes en el año 2018 (figura 2D). En la Sierra, la hepatitis A presenta la tasa de incidencia más alta, con más de 30 casos de por cada 100 000 habitantes en los años 2015-2019 (figura 2C), mientras que ninguna de las demás ETA con agente causal identificado superó los 17 casos por cada 100 000 habitantes (figura 2). La predominancia de hepatitis A observada en la región Sierra, no se replicó en la región Costa, donde durante los años 2015, 2016, 2018 y 2020, la salmonelosis superó a la de hepatitis A (figuras 2C y D). Obviando las intoxicaciones alimentarias sin agente causal definido, ninguna de las ETA analizadas superó los 25 casos por cada 100 000 habitantes en la región Costa (figura 2). En cuanto a la incidencia de fiebre tifoidea y paratifoidea desde el año 2015 al 2020 nuevamente se observa la tasa de incidencia más alta en la Amazonía; siendo el 2017 el año con el mayor número de casos (figura 2E).

La tasa de incidencia de la sumatoria de todas las ETA según la provincia en el periodo 2015-2020 se presenta en la figura 3. Existe una marcada variabilidad a lo largo de los años en las diferentes provincias y se puede identificar las provincias responsables de las diferencias a nivel regional. En el periodo 2015-2018, las provincias de la Amazonía de Napo y Orellana presentaron consistentemente las tasas más altas por cada 100 000 habitantes. En los años 2019 y 2020, Zamora Chinchipe presentó las tasas de incidencia más altas en la región amazónica. Entre los años 2015-2017, la provincia de Imbabura fue aquella con mayor incidencia de ETA en la región Sierra, mientras qué, la provincia de Manabí presentó la mayor incidencia en la región Costa en el mismo periodo (2015-2017).

**Figura 3:** Incidencia de la suma de todas las ETAs por cada 100 000 habitantes según provincia entre el 2015 y el 1020

A. Suma de todas las ETAs; B. Otras intoxicaciones alimentarias; C. Hepatitis A; D. Salmonelosis; E: Fiebre tifoidea y paratifoidea; F. Shigelosis.

## DISCUSIÓN

Este estudio sistematizó la incidencia de ETA reportadas en el sistema de vigilancia del MSP del Ecuador durante el periodo 2015-2020. A pesar de las elevadas tasas, el problema podría ser más agudo considerando que muchos casos no buscan atención médica y, en aquellos que lo hacen, no siempre se detecta el agente causal ^4^. El subregistro afecta la comprensión del problema, y por ende el accionar de las instituciones de salud ^4^. Para garantizar la inocuidad alimentaria y evitar las ETA, la detección rápida y precisa de agentes patógenos es esencial ^3^.

En Ecuador, no existen protocolos para la detección y el tratamiento de las ETA, por lo que, es posible que existan falencias en el diagnóstico e identificación de los agentes causales, explicando la alta variabilidad de las tasas de incidencia a lo largo de los años. No existe evidencia de que los datos de vigilancia epidemiológica en Ecuador se utilicen para diseñar estrategias de prevención. Hace falta la implementación de protocolos de detección, tratamiento y seguimiento para garantizar acciones oportunas para contrarrestar brotes, potencialmente mortales para las poblaciones vulnerables ^4^^,^^13^. Si bien la vigilancia epidemiológica es un pilar fundamental, la prevención efectiva podrá ser alcanzable si convergen elementos esenciales a diferentes niveles de control e involucramiento de la sociedad, leyes y regulaciones, control del manejo de alimentos, servicios de inspección, servicios de laboratorio, monitoreo de la inocuidad alimentaria, y vigilancia epidemiológica respaldada por constante educación, comunicación y entrenamiento de los manipuladores de alimentos ^14^^,^^15^.

En general, se observó consistentemente una mayor incidencia de ETA en la Amazonía, esto puede explicarse por la alta tasa de pobreza multidimensional en la región, donde en 2020, provincias amazónicas como Napo (82,5%), Pastaza (80,1%) y Morona Santiago (81,5%) presentaron tasas significativamente mayores de pobreza que provincias de la sierra como Azuay (25,6%) y Pichincha (16,9%) ^16^^-^^18^. Por otro lado, las minorías étnicas de estratos económicos bajos son afectadas en mayor proporción por las ETA ^13^ debido a inadecuados conocimientos, actitudes y prácticas, y malas prácticas en el manejo de alimentos.

La comparación de la información epidemiológica recopilada con los datos en países de similares contextos es compleja debido al potencial subregistro de casos ^2^. Con relación a la fiebre tifoidea y paratifoidea, un estudio realizado en México durante el 2018 presentó una incidencia tres veces mayor (27,9%) en comparación con lo observado en Ecuador (8,9%) ^19^. Por otro lado, un estudio en el Departamento de Casanare en Colombia con bajos índices de pobreza y clima húmedo similar a la Amazonía reportó tasas de incidencia de hepatitis A similares a las nuestras en algunos municipios ^20^, mientras que, en comparación con Chile, las tasas de incidencia en Ecuador fueron considerablemente superiores (17 casos por 100 000 habitantes en 2018 en Chile, vs. 104 casos por 100 000 habitantes en Ecuador) ^21^. Esto demuestra la dificultad en comparar los datos en regiones de ingresos medios y bajos debido a problemas en el reporte y la atención médica oportuna en el sistema de salud ^22^.

La mayoría de los casos son reportados como «otras intoxicaciones alimentarias bacterianas» reforzando la idea de que los agentes causales no son identificados en la mayor parte de los pacientes. Por otro lado, en muchos casos los reportes acumulados semana a semana no corresponden con los datos publicados en reportes previos, indicando la posibilidad de que en algunos casos el reporte fue ingresado con retraso en el Sistema Nacional de Vigilancia. Estas falencias no son nuevas, la evidencia muestra que solamente unos pocos países en el mundo han tenido éxito en tener un registro fiable y bien documentado de ETA. Incluso en los países con un adecuado sistema de registro, muchas enfermedades no llegan a ser atendidas dado que en la mayoría de los casos los síntomas son leves ^22^.

La incidencia de hepatitis A calculada ubica a Ecuador como un país con elevada incidencia de la enfermedad ^23^^,^^24^. En adolescentes y adultos, la hepatitis A puede ocasionar alteración de los marcadores de función hepática, malestar general, vómitos, anorexia y en algunos casos la muerte (2%) ^23^. Además, los pacientes pueden tardar semanas o meses en recuperarse y reanudar sus actividades diarias, influyendo negativamente en la economía y productividad ^19^. La OMS ha estimado que el costo por la pérdida de productividad total en países de ingresos bajos y medianos en $95 200 millones de dólares anuales, mientras que el costo de tratamiento de las ETA se ha estimado en 15 000 millones ^25^.

Entre las limitaciones de este estudio, se identificaron discrepancias en los valores presentados semanalmente con los valores acumulados por lo que se tomaron los valores acumulados registrados en la última semana epidemiológica de cada año. Esto dificultó el conseguir una mayor exactitud en el cálculo de incidencias o analizar los datos semana a semana.

Nuestro análisis pone en evidencia las altas tasas de incidencia de ETA, además, existe una marcada variabilidad anual en la incidencia de las ETA en las diferentes regiones geográficas del Ecuador. Se puede concluir que las ETA representan un problema de salud pública en Ecuador que afecta mayoritariamente a la región amazónica. Hace falta evaluar y optimizar el registro de casos y el sistema de vigilancia, así como diseñar estrategias preventivas integrales con especial énfasis en la región amazónica.
